# Impact of repeated anesthesia with ketamine and xylazine on the well-being of C57BL/6JRj mice

**DOI:** 10.1371/journal.pone.0203559

**Published:** 2018-09-19

**Authors:** Katharina Hohlbaum, Bettina Bert, Silke Dietze, Rupert Palme, Heidrun Fink, Christa Thöne-Reineke

**Affiliations:** 1 Institute of Animal Welfare, Animal Behavior and Laboratory Animal Science, Department of Veterinary Medicine, Freie Universität Berlin, Berlin, Germany; 2 Institute of Pharmacology and Toxicology, Department of Veterinary Medicine, Freie Universität Berlin, Berlin, Germany; 3 Unit of Physiology, Pathophysiology and Experimental Endocrinology, Department of Biomedical Sciences, University of Veterinary Medicine, Vienna, Austria; University of Queensland, AUSTRALIA

## Abstract

Within the scope of the 3Rs of Russel and Burch, the number of laboratory animals can be reduced by repeated use of an animal. This strategy only becomes relevant, if the total amount of pain, distress or harm the individual animal experiences does not exceed the severity of a single manipulation. For example, when using imaging techniques, an animal can be examined several times during a study, but it has to be anesthetized each time imaging is performed. The severity of anesthesia is thought to be mild according to the Directive 2010/63/EU. However, the Directive does not differentiate between single and repeated anesthesia, although repeated anesthesia may have a greater impact on well-being. Hence, we compared the impact of single and repeated anesthesia (six times at an interval of three to four days) by injection of ketamine and xylazine (KX) on the well-being of adult female and male C57BL/6JRj mice. After anesthesia, well-being of mice was assessed according to a protocol for systematic assessment of well-being including nesting, the Mouse Grimace Scale (MGS), a test for trait anxiety, home cage activity, and the rotarod test for motor activity, food intake, and body weight, as well as corticosterone (metabolite) analysis. Repeated anesthesia increased the MGS in mice of both sexes and caused short-term effects on well-being of female mice in the immediate post-anesthetic period, indicated by longer lasting effects on trait anxiety-related behavior. However, corticosterone metabolite concentrations suggested that mice habituated to the stress induced by repeated KX administration. Hence, the mildly negative effects on well-being of repeated KX anesthesia do not seem to accumulate over time using the respective regimen. However, further observations for severity classification are warranted in order to more specifically determine the duration of mild distress and trait anxiety.

## Introduction

The EU Directive 2010/63 “represents an important step towards achieving” the ultimate goal to phase out all animal experimentation [1]. At the same time, the Directive acknowledges that, for the time being, animal experiments are still necessary to protect human and animal health and to maintain an intact environment. Therefore, the Directive aims at improving the welfare of those animals which are still necessary to be used and at firmly anchoring the 3Rs (replace, reduce, refine) of Russel and Burch (1959) in the EU legislation. The Directive stipulates the application of the 3Rs in total [2], i.e. not only to replace animal experiments, but also to reduce the number of animals and to refine the animal experiments which are still indispensable.

One strategy to reduce the total animal number is their repeated use in the course of an experiment. This approach has been applied, for example, in studies including imaging techniques [3]. The great benefit of imaging studies with respect to animal welfare is their non-invasiveness, although tracer injection may be required prior to imaging. Instead of euthanizing several animals at different time points for further investigations, an animal can be examined several times over the course of an experiment and, thus, serves as its own control. Thereby, the progress of diseases can be monitored in one single animal. However, in terms of the 3Rs, the advantage of repeated animal use only becomes relevant if the total amount of pain, distress, and harm the single animal experiences does not accumulate and, hence, exceed the degree of severity of each single manipulation. Therefore, the reduction of the animal number for a project should not be performed on the expense of the well-being of a single animal. Balancing those values has to be carried out for each individual procedure.

In imaging studies, animals are generally anesthetized to avoid movement-associated artifacts. Hence, animals exposed to an imaging procedure several times will be repeatedly anesthetized. According to Annex VIII of the EU Directive 2010/63, anesthesia is generally classified as mild (i.e. anesthesia is likely to cause short-term mild pain, suffering or distress). However, the Directive does not distinguish between single and repeated anesthesia.

We previously showed in C57BL/6JRj mice that repeated exposure to isoflurane, the most commonly used volatile anesthetic in laboratory rodents, caused slightly more distress and impaired well-being predominantly in the immediate post-anesthetic period [4]. The advantages of volatile anesthetics over injectables are an enhanced controllability, since they are excreted by the lung and undergo almost no metabolization [5]. However, anesthesia with inhalant gases requires specific equipment, which can interfere with the experiment, e.g. when procedures are performed in a biological safety cabinet in barrier facilities [6–8]. Furthermore, the use of inhalation anesthesia may interfere with the study design or objective. If volatile anesthetics cannot be applied, injection anesthesia, such as the combination of ketamine and xylazine, is predominantly used in mice and rats [9].

Ketamine is an N-methyl-D-aspartate (NMDA) receptor antagonist and induces a dissociative anesthetic state, calaleptic sedation, and analgesia [10–13]. High dose rates are required to achieve surgical tolerance, which can be accompanied by undesirable effects such as high skeletal muscle tonus, sustained laryngeal and pharyngeal reflexes, salivary and bronchial secretions, as well as the risk of respiratory depression or arrest. Moreover, the cardiovascular system is stimulated [10–13]. In the recovery period, hallucinations can occur [10]. Ketamine disturbs the circadian rhythm [14] and has antidepressant-like properties [15], although long-term administration of ketamine can result in sex-dependent effects on anxiety-related behavior [16]. For anesthesia, ketamine is combined with an α2-adrenergic agonist such as xylazine, which provides sedation, mild to moderate analgesia, and muscle relaxation [9, 10]. Undesirable effects of xylazine are cardiovascular and respiratory depression [9, 10], hyperglycemia [17], and marked diuresis [18]. The properties of ketamine and xylazine (KX) complement each other well. The KX combination achieves surgical tolerance, reduces the high ketamine-induced skeletal muscle tonus, and diminishes its stimulation of the cardiovascular system. However, KX also causes hypotension and hypoventilation [10, 19].

The main advantage of injectable anesthetics is that no extra equipment is required [20]. However, due to the high distribution and metabolization of KX, recovery takes longer than that from volatile anesthetics [5, 21, 22]. Furthermore, the procedure of KX anesthesia includes restraining and injection stress, which in turn can influence the well-being of mice [23]. Due to the different pharmacologic properties and route of administration, the severity degree of repeated anesthesia with injectable drugs such as KX may differ from that induced by volatiles like isoflurane. Little is known about the effects of repeated KX administration on well-being and stress levels in mice. A single intraperitoneal injection can cause short-term mild pain, distress, and tissue damage [24, 25] which may potentiate over a series of injections. Post-anesthetic distress can lead to loss of body weight [26–28] and an elevated hypothalamic-pituitary-adrenal (HPA) axis activity [29]. Moreover, if mice are repeatedly anesthetized with KX during early development, motor learning and learning-dependent dendritic spine plasticity will be impaired later in life [30]. Physiological parameters such as heart rate and blood pressure are not affected compared to a single KX anesthesia. However, repeated KX anesthesia reduces sleeping time and can fail to induce surgical tolerance in rats [28, 31]. Although these findings point towards increased distress and reduced well-being in mice after repeated KX anesthesia, a systematic assessment of well-being is still lacking.

Hence, the aim of this study was to analyze the impact of repeated KX anesthesia on well-being and distress compared to single KX exposure using adult female and male C57BL/6JRj mice. We adapted our protocol of systematic well-being assessment for procedures using general anesthesia [32], which we had previously used for severity classification of repeated isoflurane anesthesia in mice [4]. In the present study, we combined behavioral observations of facial expression using the Mouse Grimace Scale (MGS), nest building, anxiety related-behavior, and activity with quantitative measures such as body weight, food intake, and stress hormone (metabolite) levels in feces and hair. Furthermore, phases of anesthesia and vital parameters were recorded. The insights of our investigation shall contribute to the refinement of anesthesia in laboratory mice.

## Methods

### Ethics statement

The study was performed according to the guidelines of the German Animal Welfare Act and the Directive 2010/63/EU for the protection of animals used for scientific purposes. Maintenance of mice and all animal experimentation were approved by the Berlin State Authority (“Landesamt für Gesundheit und Soziales”, permit number: G0053/15). Sample size calculation (primary outcome measure: effect of the anesthesia regime on fecal corticosterone metabolites) was performed to determine the number of animals necessary, as described previously [32]: n ≥ 2 × (s/μ_1_- μ_2_)^2^ × (z_α_ + z_β_)^2^; μ_1_- μ_2_ refers to the difference between population, i.e. at which power and sample size calculations are performed (α = 5%, β = 80%); z_α_ = 1.96 and z_β_ = 0.84 are the quantiles of the standard normal distribution.

Following refinement measures were executed: animals were closely monitored after anesthesia. For group-housed mice (i.e. females), the duration of single housing required to measure specific parameters, i.e. nest building, home cage activity, food intake, and fecal corticosterone metabolites (FCM), was kept to a minimum. After the experiment, female mice were rehomed and male mice were used for educational purposes.

### Animals and handling methods

A total number of 33 adult female and 31 adult male C57BL/6JRj mice obtained from Janvier Labs (Saint-Berthevin Cedex, France) at 10–13 weeks of age were used. This strain was chosen since C57BL/6JRj mice are the most commonly used laboratory mice. The mice were assigned to six study groups by simple randomisation: control ♀ (n = 7), control ♂ (n = 6), single anesthesia ♀ (n = 13), single anesthesia ♂ (n = 13), repeated anesthesia ♀ (n = 13), repeated anesthesia ♂ (n = 12; one mouse had to be euthanized due to respiratory depression after the sixth anesthesia). Female mice were group-housed with three to five mice in Makrolon type IV cages (55 × 33 × 20 cm). Male mice had to be single-housed in Makrolon type III cages (42 × 26 × 15 cm) during the entire study due to aggressive behavior toward conspecifics. The cages contained fine wooden bedding material (LIGNOCEL® 3–4 S, J. Rettenmaier & Söhne GmbH + Co. KG, Rosenberg, Germany) and nestlets (Ancare, UK agents, Lillico, United Kingdom). A red plastic house (length: 100 mm, width: 90 mm, height: 55 mm; ZOONLAB GmbH, Castrop-Rauxel, Germany) and metal tunnels (length: 125 mm, diameter: 50 mm; one tunnel in Makrolon type III cages, two tunnels in Makrolon type IV cages) were provided as cage enrichment. The animals were maintained under standard conditions (room temperature: 22 ± 2°C; relative humidity: 55 ± 10%) on a light:dark cycle of 12:12 h of artificial light with a 5 min twilight transition phase (lights on from 6:00 a.m. to 6:00 p.m.). The mice were fed pelleted mouse diet ad libitum (Ssniff rat/mouse maintenance, Spezialdiäten GmbH, Soest, Germany) and had free access to tap water.

In order to prevent influence due to stress caused by male persons, both the technician and veterinarian were female [33]. One week prior to experiments, the mice were habituated to handling by combined tunnel and cup handling. The mice were carefully caught in a tunnel belonging to the standard enrichment and then transferred to the experimenter’s hands. This method is known to cause less anxiety than picking up by the tail [34].

### Test schedule

**Fig 1:**
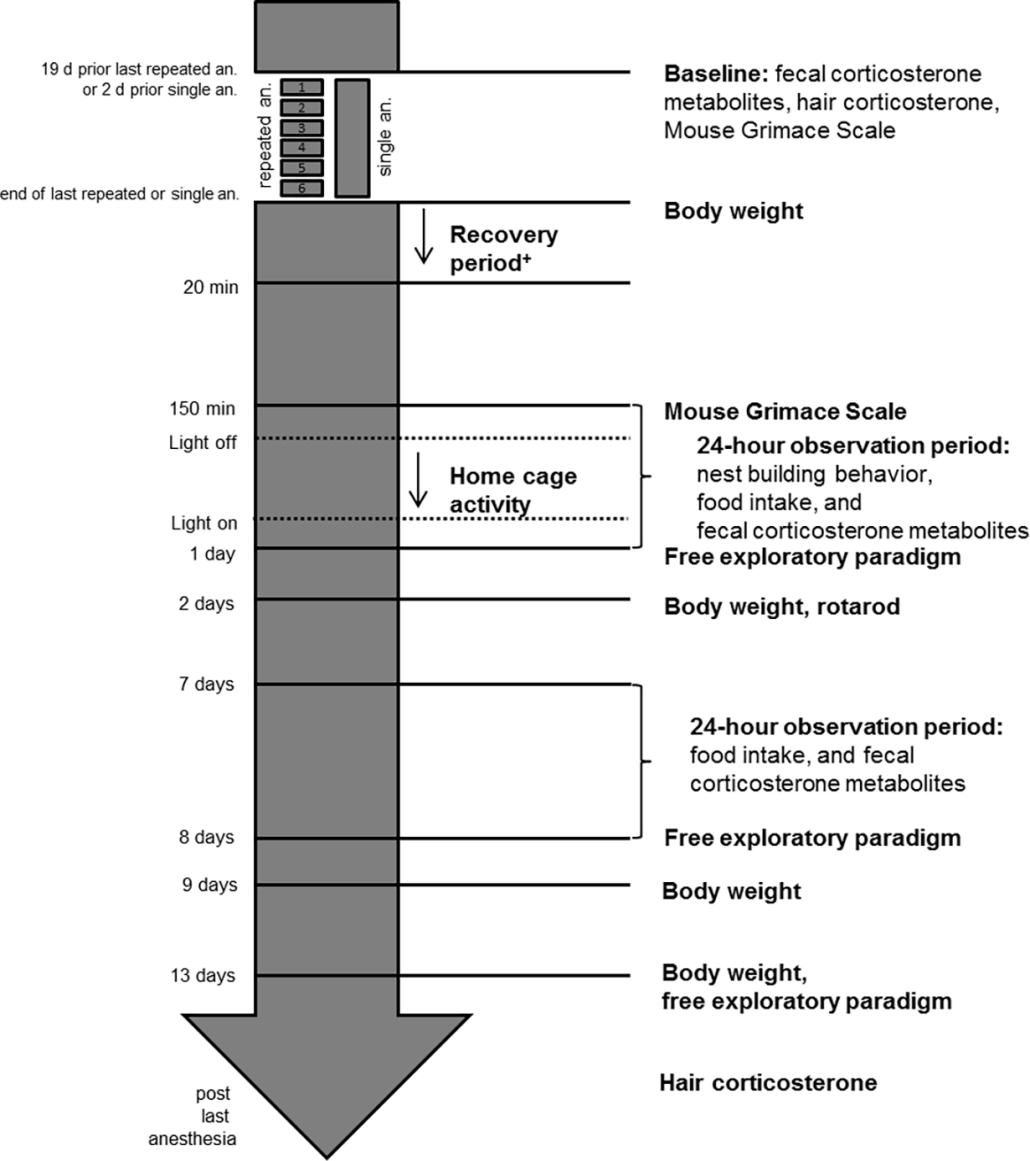
Flow chart of the test schedule. ^+^ Duration of resting [s], duration of locomotion [s], number of falls [n], and duration of episodes of rapid jerky dorsal-ventral head movements [s]; an.: anesthesia. This figure has been modified from Hohlbaum et *al*. [4, 32].

In the following section, the test schedule is described in brief. Detailed information on materials and methods can be found in the respective sections. The test schedule was adapted from our previous study [4, 32] and is outlined in Fig 1. In the beginning of the experiment, photos for the baseline MGS scores were taken and samples for both baseline values of FCM and hair corticosterone were collected.

Anesthesia was performed as follows: mice in the single anesthesia-group were anesthetized once with a single KX injection, whereas mice in the repeated anesthesia-group were anesthetized six times at three to four day intervals over a period of three weeks.

After KX administration, the onset of the righting reflex loss was recorded and the pedal withdrawal reflex of the front and hind paws as well as the lid reflex were tested every min. After the pedal withdrawal reflexes were lost, all reflexes were monitored every 10 min and vital parameters (i.e. heart rate, oxygen saturation, respiratory rate, and body temperature) were carefully monitored and recorded every 20 min. The end of anesthesia was defined as the time point at which the first forward movement was observed. Mice received only one dose of KX–even if they did not reach surgical tolerance. When mice showed constant forward movements for about two to three min, they were transferred to a custom-made glass box (22 × 29 × 39 cm, consisting of three white walls and one clear wall, with 0.5 cm bedding material including soiled bedding) and their behavior was monitored for 20 min. Photographs of the mouse faces for the MGS were taken at 150 min after awakening. Afterwards, all mice were transferred into a new Makrolon type III cage and single-housed for a period of 24 h, allowing for food intake measurement and collection of fecal samples for FCM analysis. Moreover, home cage activity was recorded for 12 h during the dark period and nest building was evaluated in the morning of the following day. Measurement of food intake and FCM analyses were repeated one week later. In order to observe a change in anxiety-related and exploratory behavior over time, the free exploratory paradigm was performed at day 1, 8, and 13 after the last anesthesia. Two days after the last anesthesia, the rotarod test for motor coordination and balance was conducted and another photograph for the MGS was taken. At the end of the experiment, a sample of regrown hair was collected from the same area from which a hair sample for corticosterone measurement had been taken prior to the study. Mice were weighed regularly during the entire study period.

### Anesthesia

#### Anesthesia protocol

A stock solution was prepared in a syringe including 160 μL Ketavet® 100 mg/mL (Zoetis Deutschland GmbH, Berlin, Germany), 160 μL Rompun® 2% (Bayer Vital GmbH, Leverkusen, Germany), and 1680 μL physiologic saline solution. A dosage of 80 mg/kg ketamine and 16 mg/kg xylazine [35], warmed to body temperature, was administered intraperitoneally at a volume of 10 μL/g body weight using 27 ¾ Gauge needles. Sites of injection were alternated to prevent tissue damage [36]. After the administration of KX, the mouse was transferred to a Makrolon Typ III cage, which was placed on a heating pad. When the mouse lost its righting reflex, it was laid in abdominal position on a heating pad and, subsequently, the loss of pedal withdrawal reflex of both front and hind paws as well as lid reflex was carefully monitored. Artificial tears (Artelac® Splash MDO®, Bausch & Lomb GmbH, Berlin, Germany) were administered to both eyes to protect them from drying out.

Control mice did not receive any treatment since we intended to investigate the impact of the entire KX injection anesthesia procedure, also including restraint and injection besides pharmacological effects. If control mice had received an injection of sodium chloride, we could have only examined the pharmacological effect of KX, which was not the main focus of this study.

#### Anesthesia phases

Anesthesia was divided in five anesthetic stages [37]: 1) induction of anesthesia starting with injection of KX and ending with the loss of righting reflex. 2) Non-surgical-tolerance was defined as the time from loss of righting reflex to loss of pedal withdrawal and lid reflex. 3) Surgical tolerance began with the loss of all reflexes examined and ended with the regain of pedal withdrawal reflex (during surgical tolerance, reflexes were tested every 10 min and the time of regaining the pedal withdrawal reflex was noted). 4) Then, the wake-up period followed (after the first reflex was regained, reflexes were tested approximately every 2 min) and ended after the first forward movement (latency to the first forward movement after anesthesia [s]). 5) The recovery period was defined as the time during which mice showed constant forward movements for about 2–3 min until 20 min after anesthesia.

#### Vital parameters

During anesthesia, vital parameters (respiratory rate, heart rate, oxygen saturation, and body temperature) were carefully monitored at 20 min and 40 min after injection. Respiratory rate was counted for 15 sec and calculated for 1 min [breaths per min]. A pulse oximeter (MouseOx, STARR Life Sciences® Corp., Oakmont, PA, USA), attached to the shaved left hind leg, was used to measure heart rate [beats per min] and oxygen saturation [%]. Body temperature was measured using a rectal probe.

### Recovery period

The mice were video-recorded for 20 min after the first forward movement in the custom-made photography cube. Duration of resting (no movement of the limbs and the head) [s], duration of locomotion [s], number of falls (while walking the mouse loses balance and falls on its left or right side; falls are defined as a pattern of ataxic gait [38]) [n], and duration of episodes of rapid jerky dorsal-ventral head movements [s] with the body remaining stationary (these movements can be defined as stereotypic behavior [39]) were manually measured with ethological analyses software (Etholog version 2.2.5 [40]).

### Behavioral parameters and stress hormone (metabolite) analyses

The MGS, nest building, home cage activity during the dark period, the free exploratory paradigm for trait anxiety related behavior, the rotarod test for motor coordination and balance, food intake/body weight, FCM concentrations for acute stress during the 24-h post-anesthetic period, and hair corticosterone concentrations for chronic stress were investigated as described in S1 Methods and in our previous studies [4, 32].

### Statistical analysis

Statistical analysis was performed with IBM SPSS Version 23 (IBM Corporation, Armonk, NY, USA). Explorative data analysis and tests for normality were performed for each parameter. First, differences between female groups (control, single anesthesia, repeated anesthesia), secondly, differences between male groups (control, single anesthesia, repeated anesthesia), and, thirdly, sex differences (female versus male control, female versus male single anesthesia, female versus male repeated anesthesia) were analyzed using the respective test indicated in results section (repeated measures ANOVA, One-way ANOVA, Kruskal-Wallis-Test, Mann-Whitney-U-Test or unpaired Student t-test). Differences were considered significant at p < 0.05. Fleiss kappa was calculated using Microsoft Excel (2013). It is important to note that mice, which did not reach surgical tolerance, were not excluded from statistical analyses. One male mouse of the repeated anesthesia group was excluded from the statistics since it had to be euthanized after the sixth anesthesia due to dyspnea.

## Results

### Anesthesia

#### Phases of anesthesia

**Comparison of females:** Repeated anesthesia significantly shortened the overall duration of anesthesia (unpaired Student t-test: t = 5.026, df = 16.187, p < 0.001) and the duration of surgical tolerance (Mann-Whitney-U-Test: U = 16.000, p < 0.001) compared to a single anesthesia (Table 1). During the sixth anesthesia, four mice did not reach surgical tolerance.

**Table 1:** Phases of anesthesia.

Group	Induction^1^ [s]	Non-surgical tolerance^1^ [s]	Surgical tolerance^2^ [s]	Wake-up period^2^ [s]	Duration of anesthesia^2^ [s]
**Single anesthesia ♀** (n = 13)	96 ± 31	444 ± 195	2658 ± 705	1507 ± 426^#^	4609 ± 658
**Repeated anesthesia ♀** (n = 13)	93 ± 10	594 ± 403	1394 ± 846***	1578 ± 273	3612 ± 279***^,^^###^
**Single anesthesia ♂** (n = 13)	108 ± 28	595 ± 287	2471 ± 1000	1991 ± 636	5057 ± 474
**Repeated anesthesia ♂** (n = 12)	90 ± 26	817 ± 755	1714 ± 1120	1814 ± 830	4365 ± 517**

Data are given as mean ± standard deviation.

^1^Data were analyzed using Mann-Whitney-U-Test or

^2^unpaired Student t-test

** p < 0.01

*** p < 0.001 versus single anesthesia

^#^ p < 0.05

^###^ p < 0.001 versus corresponding ♂. ♀: females; ♂: males.

**Comparison of males:** In comparison to a single anesthesia, repeated anesthesia significantly shortened the overall duration of anesthesia (unpaired Student t-test: t = 3.494, df = 23, p = 0.002) (Table 1). Duration of surgical tolerance was also shorter in mice repeatedly anesthetized compared to mice anesthetized only once but no statistical significance was reached (Mann-Whitney-U-Test: U = 51.000, p = 0.152). During the sixth anesthesia, one mouse did not reach surgical tolerance.

**Sex differences:** Sex differences were observed after a single anesthesia with a shorter wake-up period in female mice (unpaired Student t-test: t = –2.281, df = 24, p = 0.032) and also after repeated anesthesia with a shorter overall anesthesia duration in female mice (unpaired Student t-test: t = 4.476, df = 16.624, p < 0.001) (Table 1).

#### Vital parameters

The measurements of heart rate, oxygen saturation, respiratory rate, and body temperature at 20 min and 40 min after injection were averaged (for the groups with repeated anesthesia, values of the sixth anesthesia used) (Table 2).

**Table 2:** Vital parameters.

Group	Heart rate [bpm]	Oxygen saturation [%]	Respiratory rate [brpm]	Body temperature [°C]
**Single anesthesia ♀** (n = 13)	280 ± 32	77.5 ± 4.3^#^	147 ± 19	36.2 ± 0.5
**Repeated anesthesia ♀** (n = 13)	296 ± 18	76.6 ± 4.4	152 ± 14	36.3 ± 0.3
**Single anesthesia ♂** (n = 13)	285 ± 25	73.4 ± 4.9	151 ± 20	36.4 ± 0.4
**Repeated anesthesia ♂** (n = 12)	297 ± 30	75.4 ± 4.9	157 ± 12	36.4 ± 0.2

Data are given as mean ± standard deviation. bmp: beats per min; brpm: breaths per min. Data were analyzed using unpaired Student t-test:

^#^ p < 0.05 versus corresponding ♂. ♀: females; ♂: males.

**Comparison of females:** Vital parameters did not differ between single and repeated anesthesia (heart rate: unpaired Student t-test: t = –1.589, df = 18.815, p = 0.129; oxygen saturation: unpaired Student t-test: t = 0.562, df = 24, p = 0.580; respiratory rate: unpaired Student t-test: t = –0.649, df = 24, p = 0.522; body temperature: unpaired Student t-test: t = –0.118, df = 24, p = 0.907) (Table 2).

**Comparison of males:** Vital parameters did not differ between single and repeated anesthesia (heart rate: unpaired Student t-test: t = –1.044, df = 23, p = 0.307; oxygen saturation: unpaired Student t-test: t = –1.006, df = 23, p = 0.325; respiratory rate: unpaired Student t-test: t = –0.931, df = 23, p = 0.362; body temperature: unpaired Student t-test: t = –0.222, df = 18.449, p = 0.827) (Table 2).

**Sex differences:** There was a significant difference in oxygen saturation only. Oxygen saturation was higher in females than it was in males during single anesthesia (unpaired Student t-test: t = 2.279, df = 24, p = 0.032) (Table 2).

### Recovery period

**Comparison of females:** During the recovery period, the number of falls was more frequently observed after repeated anesthesia than after single anesthesia (Mann-Whitney-U-Test: U = 133.500, p = 0.010) (Table 3). Furthermore, the duration of episodes of rapid jerky dorsal-ventral head movements was longer after repeated anesthesia compared to single anesthesia but statistical significance was not reached (Mann-Whitney-U-Test: U = 104.000, p = 0.336).

**Table 3:** Recovery period.

Group	Duration of resting^2^ [s]		Duration of locomotion^2^ [s]		Number of falls^1^ [n]		Duration of episodes of rapid jerky dorsal-ventral head movements^1^ [s]	
	M	IQR	M	IQR	M	IQR	M	IQR
**Single anesthesia ♀** (n = 13)	453	318–679	468	251–606	4	1–8	160	96–299
**Repeated anesthesia ♀** (n = 13)	298	231–469	540^#^	439–594	25*	15–56	300	116–387
**Single anesthesia ♂** (n = 13)	490	341–692	472	281–752	1	0–10	144	75–212
**Repeated anesthesia ♂** (n = 12)	417	244–601	403	255–490	16**	10–34	382***	202–500

Data are given as median (M) and interquartile range (IQR). Data were analyzed using

^1^Mann-Whitney-U-Test or

^2^unpaired Student t-test.

* p < 0.05

** p < 0.01

*** p < 0.001 versus a single anesthesia

^#^ p < 0.05 versus corresponding ♂. ♀: females; ♂: males.

**Comparison of males:** Repeated anesthesia increased both number of falls (Mann-Whitney-U-Test: U = 135.000, p = 0.001) and duration of episodes of rapid jerky dorsal-ventral head movements (Mann-Whitney-U-Test: U = 139.000, p < 0.001) in comparison to single anesthesia (Table 3).

**Sex differences:** Sex differences were detected for the duration of locomotion, which was higher in female mice than it was in male mice after repeated anesthesia (unpaired Student t-test: t = –2.628, df = 23, p = 0.015) (Table 3).

### Behavioral parameters and stress hormone (metabolite) analyses

#### Mouse Grimace Scale (MGS)

For the MGS difference scores, Fleiss’ kappa (κ = 0.34) indicated fair agreement [41] between the 4 independent scorers, when MGS difference scores were divided into the following categories: 1) MGS difference score ≤ 0.5, 2) 0.5 < MGS difference score ≤ 1.0, 3) 1.0 < MGS difference score ≤ 1.5, 4) 1.5 < MGS difference score ≤ 2.0.

**Fig 2:**
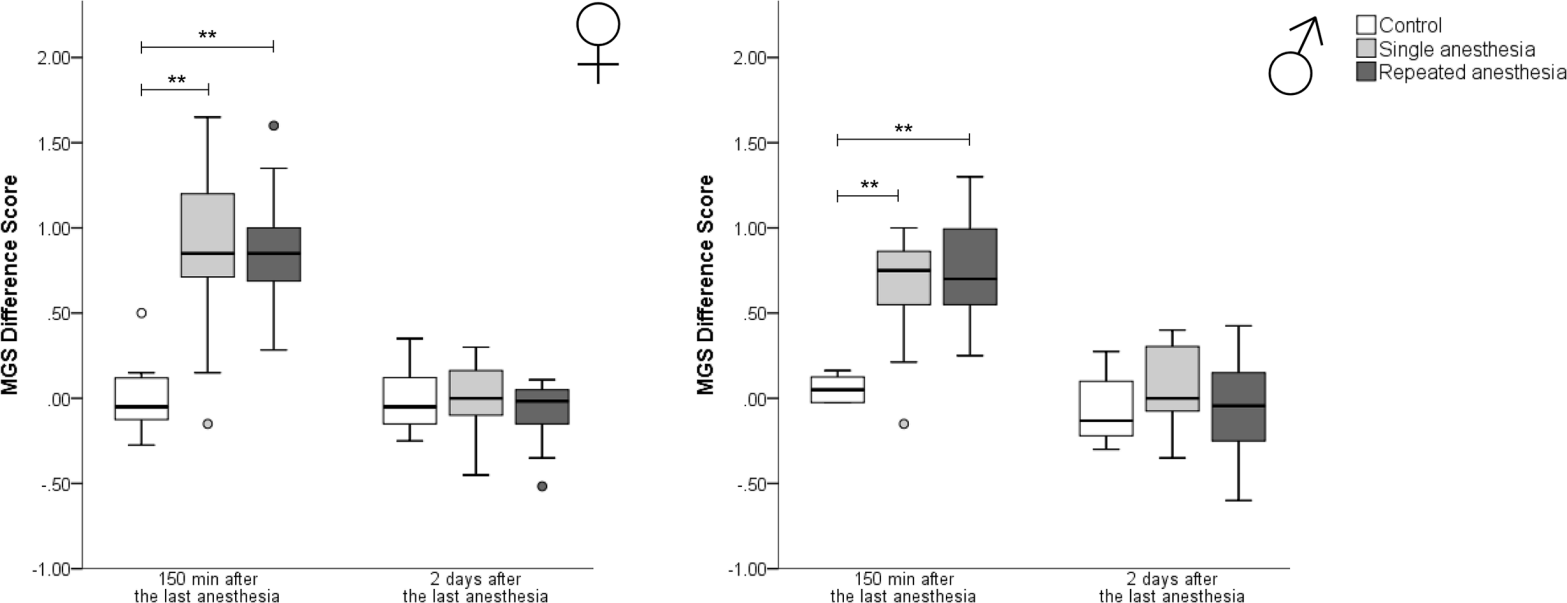
Mouse Grimace Scale difference scores. MGS, Mouse Grimace Scale; ♀: females; ♂: males. Data are presented as boxplot diagrams: the box represents the interquartile range (IQR), box edges are the 25^th^ and 75^th^ quartile. The whiskers represent values which are not greater than 1.5 × IQR. Dots are outliers with values between 1.5‒3.0 × IQR. Grey asterisks are outliers with values greater than 3.0 × IQR. Data were analyzed using Kruskal-Wallis-Test with post-hoc Dunn-Bonferroni test or, for sex differences, Mann-Whitney-U-Test ** p < 0.01 versus control). (A) Control ♀: n = 7, single anesthesia ♀: n = 13 at 150 min (n = 9 at day two; at this study day, four mice of the single anesthesia group were excluded from statistics due to technical malfunction of the camera), repeated anesthesia ♀: n = 13. (B) Control ♂: n = 6, single anesthesia ♂: n = 13, repeated anesthesia ♂: n = 12.

**Comparison of females:** Both single (Kruskal-Wallis-Test: z = 3.271, p = 0.003) and repeated anesthesia (Kruskal-Wallis-Test: z = 3.331, p = 0.003) caused significant higher MGS difference scores versus control at 150 min after the last anesthesia (Fig 2A). Two days after the last anesthesia, the MGS difference scores no longer differed from control animals (Kruskal-Wallis-Test: Chi^2^ = 0.633, df = 2, p = 0.701).

**Comparison of males:** Both single (Kruskal-Wallis-Test: z = 3.150, p = 0.005) and repeated anesthesia (Kruskal-Wallis-Test: z = 3.264, p = 0.003) significantly increased MGS difference scores versus control at 150 min after the last anesthesia (Fig 2B). Two days after the last anesthesia, there were no differences between the groups anymore (Kruskal-Wallis-Test: Chi^2^ = 1.379, df = 2, p = 0.502).

**Sex differences:** No sex differences were found at 150 min (Mann-Whitney-U-Test: control: U = 24.000, p = 0.731; single anesthesia: U = 55.000, p = 0.139; repeated anesthesia: U = 59.000, p = 0.320) or two days (Mann-Whitney-U-Test: control: U = 16.500, p = 0.534; single anesthesia: U = 66.500, p = 0.601; repeated anesthesia: U = 83.500, p = 0.769) after the last anesthesia.

#### Nest building

**Comparison of females:** No significant differences in the nest scores were found between control, single anesthesia, and repeated anesthesia (Kruskal-Wallis-Test: Chi^2^ = 4.331, df = 2, p = 0.115) (Table 4).

**Table 4:** Nest building.

Group	M	IQR
**Control ♀** (n = 7)	5.0	4.0–5.0
**Single anesthesia ♀** (n = 13)	4.0	2.0–4.5
**Repeated anesthesia ♀** (n = 13)	3.0	3.0–4.0
**Control ♂** (n = 6)	4.5	3.5–5.0
**Single anesthesia ♂** (n = 13)	5.0	3.0–5.0
**Repeated anesthesia ♂** (n = 12)	2.5	2.0–4.0

Data are given as median (M) and interquartile range (IQR). Data were analyzed using Kruskal-Wallis-Test with post-hoc Dunn-Bonferroni test or, for sex differences, Mann-Whitney-U-Test. ♀: females; ♂: males.

**Comparison of males:** There were no differences between control, single anesthesia, and repeated anesthesia (Kruskal-Wallis-Test: Chi^2^ = 4.266, df = 2, p = 0.118) (Table 4).

**Sex differences:** The Mann-Whitney-U-Test revealed no sex differences (control: U = 18.500, p = 0.731; single anesthesia: U = 107.000, p = 0.264; repeated anesthesia: U = 59.500, p = 0.320).

#### Home cage activity

**Comparison of females:** On day one after the last anesthesia, Kruskal-Wallis-Analysis revealed no differences in the home cage activity during the dark period between control animals and the two treatment groups (Chi^2^ = 0.498, df = 2, p = 0.780) (Table 5).

**Table 5:** Home cage activity during the dark period (one day after the last anesthesia).

Group	AUC of home cage activity [impulses/10min]	
	Median	IQR
**Control ♀** (n = 6)	4.5M	2.8M–6.5M
**Single anesthesia ♀** (n = 12)	5.5M	3.1M–7.7M
**Repeated anesthesia ♀** (n = 9)	5.6M	4.0M–7.3M
**Control ♂** (n = 6)	6.2M	5.3M–7.7M
**Single anesthesia ♂** (n = 13)	3.7M	3.3M–6.2M
**Repeated anesthesia ♂** (n = 9)	4.8M	3.4M–5.9M

Data are given as median and interquartile range (IQR). AUC: area under the curve; M: million. Data were analyzed using the Kruskal-Wallis-Test with post-hoc Dunn-Bonferroni test or, for sex differences, Mann-Whitney-U-Test. ♀: females; ♂: males.

Due to technical malfunction of the InfraMot system, one mouse of the control ♀ group, one mouse of the single anesthesia ♀ group, four mice of the repeated anesthesia ♀ group, and three mice of the repeated anesthesia ♂ group were excluded from statistics.

**Comparison of males:** There was no significant difference in the home cage activity during the dark period between control mice and the two anesthesia groups (Chi^2^ = 5.271, df = 2, p = 0.072) (Table 5).

**Sex differences:** Mann-Whitney-U-Test showed no sex differences in the home cage activity (control: U = 28.000, p = 0.132; single anesthesia: U = 61.000, p = 0.376; repeated anesthesia: U = 29.000, p = 0.340).

#### Rotarod test

**Comparison of females:** In the rotarod test, Kruskal-Wallis-Analysis revealed no significant differences in the latency to fall between the control, single anesthesia, and repeated anesthesia (Chi^2^ = 0.722, df = 2, p = 0.697) (Table 6).

**Table 6:** Physical fitness (two days after the last anesthesia).

Group	Latency to fall in the rotarod test [s]	
	2 days after the last anesthesia	
	M	IQR
**Control ♀** (n = 7)	300	237–300
**Single anesthesia ♀** (n = 13)	300	210–300
**Repeated anesthesia ♀** (n = 13)	280	178–300
**Control ♂** (n = 6)	300	249–300
**Single anesthesia ♂** (n = 13)	300	217–300
**Repeated anesthesia ♂** (n = 12)	261	130–300

Data are given as median (M) and interquartile range (IQR). Data were analyzed using the Kruskal-Wallis-Test with post-hoc Dunn-Bonferroni test or, for sex differences, Mann-Whitney-U-Test. ♀: females; ♂: males.

**Comparison of males:** In male mice, the latency to fall in the rotarod test did not significantly differ between the three groups (Chi^2^ = 2.295, df = 2, p = 0.317) (Table 6).

**Sex differences:** No sex differences were found (control: U = 21.000, p = 1.000; single anesthesia: U = 87.000, p = 0.920; repeated anesthesia: U = 64.000, p = 0.470).

#### Free exploratory paradigm

In the free exploratory paradigm for trait anxiety-related and exploratory behavior, all mice, independently of the group, explored the cage top.

**Fig 3:**
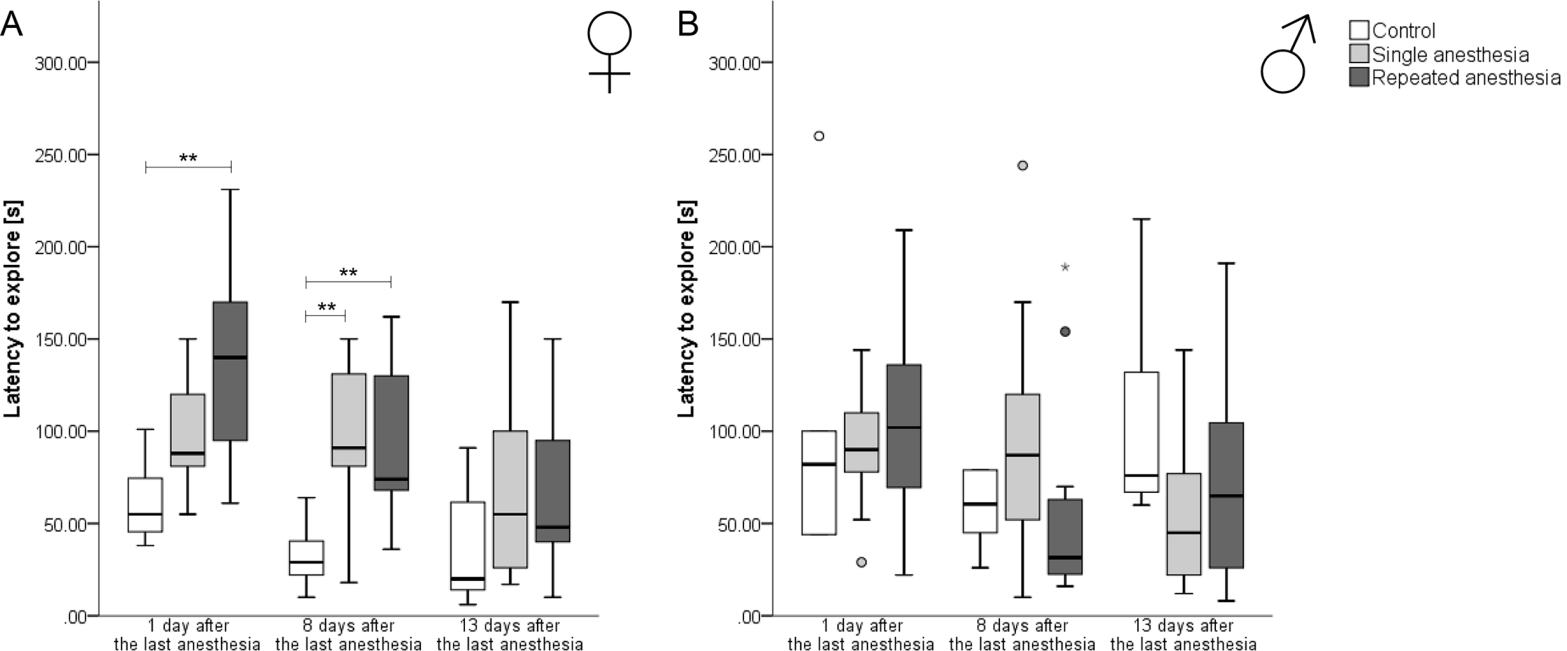
Latency to explore in the free exploratory paradigm for trait anxiety-related and exploratory behavior. ♀: females; ♂: males. Data are presented as boxplot diagrams: the box represents the interquartile range (IQR), box edges are the 25^th^ and 75^th^ quartile. The whiskers represent values which are not greater than 1.5 × IQR. Dots are outliers with values between 1.5 ‒ 3.0 × IQR. Grey asterisks are outliers with values greater than 3.0 × IQR. Data were analyzed using the Kruskal-Wallis-Test with post-hoc Dunn-Bonferroni test or, for sex differences, Mann-Whitney-U-Test (** p < 0.01 versus repeated anesthesia). (A) Control ♀: n = 7, single anesthesia ♀: n = 13, repeated anesthesia ♀: n = 13. (B) Control ♂: n = 6, single anesthesia ♂: n = 13, repeated anesthesia ♂: n = 12.

**Comparison of females:** Animals repeatedly treated with KX showed a significantly higher latency to explore the top of the cage one day after the last anesthesia than control mice (Kruskal-Wallis-Test: Chi^2^ = 13.670; df = 2, z = 3.671, p = 0.001). This effect was still present eight days after anesthesia (Kruskal-Wallis-Test: Chi^2^ = 11.673; df = 2, repeated anesthesia versus control: z = 3.036, p = 0.007). In addition, on day eight animals exposed only to a single KX anesthesia also took significantly longer to explore the outside of the cage (single anesthesia versus control: z = 3.164, p = 0.005). On day 13 after the last anesthesia, there were no significant differences in the latency to explore between the three groups anymore (Kruskal-Wallis-Test: Chi^2^ = 3.429, df = 2, p = 0.180) (Fig 3A).

**Comparison of males:** There were no significant differences in the latency to explore between the groups on day one (Kruskal-Wallis-Test: Chi^2^ = 0.974, df = 2, p = 0.614), 8 days (Kruskal-Wallis-Test: Chi^2^ = 4.805, df = 2, p = 0.091) and at 13 days (Kruskal-Wallis-Test: Chi^2^ = 0.258, df = 2, p = 0.258) after the last anesthesia (Fig 3B).

**Sex differences:** On day eight after the last anesthesia, there was a significant difference in the latency to explore in mice receiving repeated anesthesia, with a lower latency to explore in male mice *versus* female mice (Mann-Whitney-U-Test: U = 33.500, p = 0.014).

#### Food intake

**Comparison of females:** One-way ANOVA revealed no significant difference in food intake between the three groups on day one (One-way ANOVA: F = 1.039, between-group df = 2, within-group df = 30, p = 0.366) and day eight (F = 0.221, between-group df = 2, within-group df = 30, p = 0.803) after the last anesthesia (Table 7).

**Table 7:** Food intake.

Group	Food Intake[g/g body weight]	
	1 day after last anesthesia	8 days after last anesthesia
**Control ♀** (n = 7)	0.22 ± 0.05	0.18 ± 0.08
**Single anesthesia ♀** (n = 13)	0.20 ± 0.04^##^	0.20 ± 0.06
**Repeated anesthesia ♀** (n = 13)	0.19 ± 0.04	0.20 ± 0.07
**Control ♂** (n = 6)	0.17 ± 0.03	0.21 ± 0.03
**Single anesthesia ♂** (n = 13)	0.15 ± 0.03	0.19 ± 0.05
**Repeated anesthesia ♂** (n = 12)	0.18 ± 0.04*	0.22 ± 0.04

Data are given as mean ± standard deviation. Data were analyzed using One-way ANOVA with post-hoc Dunnett-T3 (* p < 0.05 *versus* single anesthesia) or, for sex differences, unpaired Student t-Test (^##^ p < 0.01 *versus* corresponding ♂). ♀: females; ♂: males.

**Comparison of males:** There was a significant difference in food intake between single and repeated anesthesia on day one after the last anesthesia (One-way ANOVA: F = 4.292, between-group df = 2, within-group df = 28, p = 0.024; post-hoc Dunnett-T3: p = 0.031) (Table 7). Male mice receiving single anesthesia ingested significantly less food in comparison to male mice receiving repeated anesthesia. Eight days after the last anesthesia, there was no significant difference between the groups (One-way ANOVA: F = 1.739, between-group df = 2, within-group df = 28, p = 0.194).

**Sex differences:** On day one after the last anesthesia, there was no significant sex difference in food intake after repeated anesthesia (unpaired Student t-Test: t = 0.404, df = 23, p = 0.690) or between controls (unpaired Student t-Test: t = 1.980, df = 11, p = 0.073). However, after single anesthesia, males ingested significantly less food than females did (unpaired Student t-Test: t = 3.680, df = 24, p = 0.001) (Table 7).

#### Body weight

**Fig 4:**
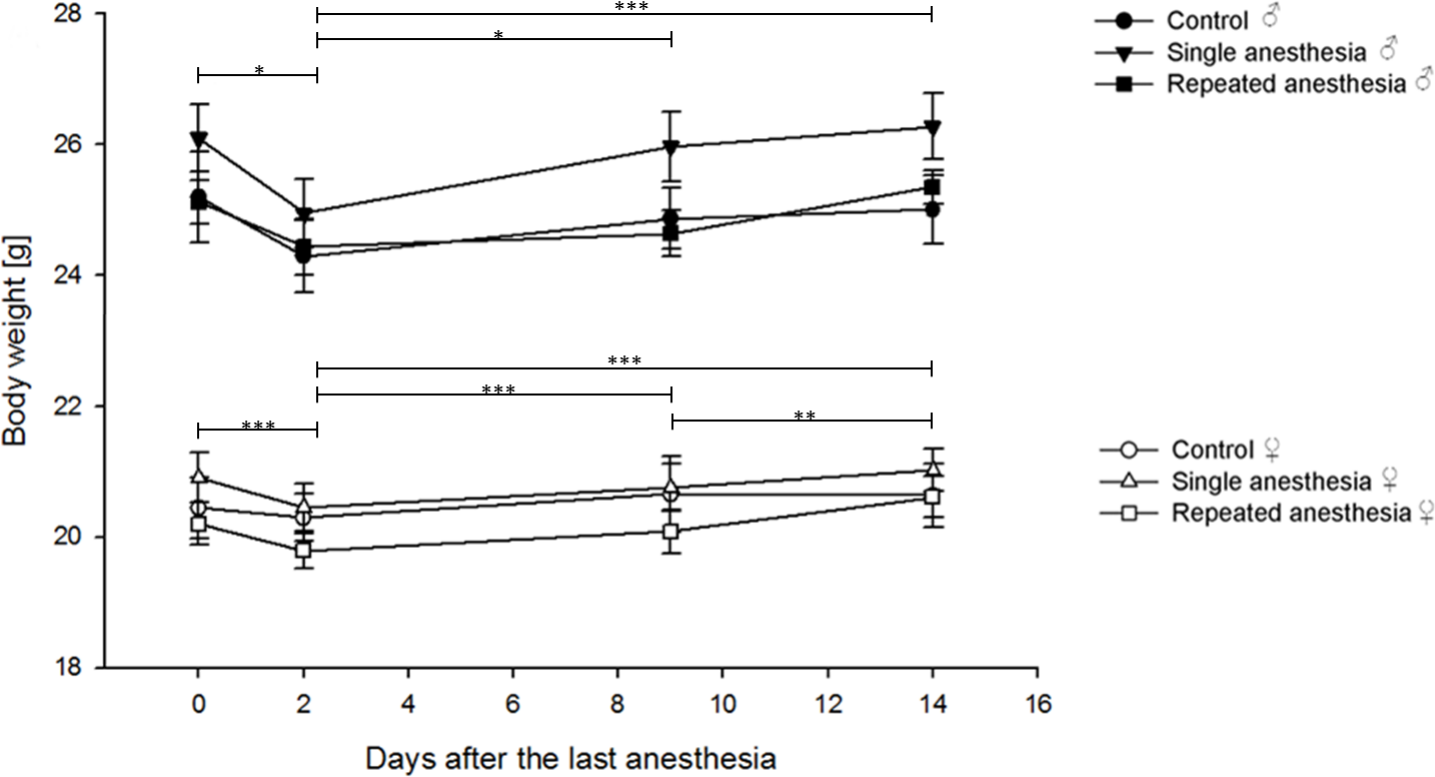
Course of body weight. Data are given as mean ± standard error. (A) Control ♀: n = 7, single anesthesia ♀: n = 13, repeated anesthesia ♀: n = 13. (B) Control ♂: n = 6, single anesthesia ♂: n = 13, repeated anesthesia ♂: n = 12. Data were analyzed using repeated measures ANOVA with group as between-subject factor (pairwise comparisons using Bonferroni correction: * p < 0.05, *** p < 0.001 versus day two; ** p < 0.01 versus day 14).

Tests of between-subject comparison indicated that the group (using repeated measures ANOVA with group as between-subject factor: females: F = 0.803, between-group df = 2, within-group df = 30, p = 0.457; males: F = 1.500, between-group df = 2, within-group df = 28, p = 0.241) had no effect on the body weight, but there was a significant time effect (females: F = 10413.101, between-group df = 2, within-group df = 30, p < 0.001; males: F = 7622.477, between-group df = 2, within-group df = 28, p < 0.001) (Fig 4).

### Corticosterone and its metabolites

For each mouse, the percentage change [%] relative to baseline was calculated with baseline values being defined as 100%.

**Comparison of females:** On day one (Kruskal-Wallis-Test: Chi^2^ = 3.682, df = 2, p = 0.159) after the last anesthesia, FCM concentrations were higher after single anesthesia, despite no statistical significance being reached between the three female groups. On day eight (Kruskal-Wallis-Test: Chi^2^ = 0.099, df = 2, p = 0.952) after the last anesthesia, female groups did not significantly differ in their FCM concentrations. Moreover, there was no statistically significant difference in hair corticosterone levels between control, single anesthesia, and repeated anesthesia (Kruskal-Wallis-Test: Chi^2^ = 4.431, df = 2, p = 0.109) (Table 8, S1 Table).

**Table 8:** Fecal corticosterone metabolites (FCM) and hair corticosterone.

Group	FCM [%]				Hair corticosterone [%]	
	1 day after the last anesthesia		8 days after the last anesthesia			
	M	IQR	M	IQR	M	IQR
**Control ♀** (n = 7)	102.74	87.05–139.52	147.02	75.57–154.88	87.37	81.45–105.83
**Single anesthesia ♀** (n = 13)	146.35^#^	105.90–187.89	138.87	109.32–157.76	71.95	10.34–88.99
**Repeated anesthesia ♀** (n = 13)	117.15	84.35–132.31	124.89	107.50–178.80	88.69	71.36–111.28
**Control ♂** (n = 6)	124.94	76.13–153.02	95.11	84.84–141.28	87.00	82.52–95.00
**Single anesthesia ♂** (n = 13)	220.49*	154.90–248.58	125.44	83.16–171.47	102.04	33.26–126.90
**Repeated anesthesia ♂** (n = 12)^**a**^	181.72	97.92–225.01	173.15	62.18–254.29	99.02	76.98–135.39

The percentage change relative to the baseline value was calculated. Baseline values are defined as 100%. Data are given as median (M) and interquartile range (IQR). FCM: fecal corticosterone metabolites. Data were analyzed using the Kruskal-Wallis-Test with post-hoc Dunn-Bonferroni test (* p < 0.05 versus control) or, for sex differences, Mann-Whitney-U-Test (^#^ p < 0.05 versus corresponding ♂). ♀: females; ♂: males.

^a^ One male mouse of the repeated anesthesia group was excluded from the statistics concerning hair corticosterone (n = 11) since too little sample material was available.

**Comparison of males:** Single anesthesia increased FCM concentrations on day one after the last anesthesia compared to control mice (Kruskal-Wallis-Test: Chi^2^ = 7.177, df = 2; single anesthesia versus control: z = 2.674, p = 0.022), whereas no significant change in FCM concentrations on day eight could be detected (Kruskal-Wallis-Test: Chi^2^ = 1.276, df = 2, p = 0.528) (Table 8). Hair corticosterone levels did not significantly differ between control, single anesthesia, and repeated anesthesia (Kruskal-Wallis-Test: Chi^2^ = 0.498, df = 2, p = 0.780) (Table 8).

**Sex differences:** One day after the single anesthesia, the percentage change relative to FCM baseline values was higher in males when compared to females (Mann-Whitney-U-Test: U = 128.000, p = 0.026) (Table 8). There were no sex differences in hair corticosterone concentrations in control mice (Mann-Whitney-U-Test: U = 21.000, p = 1.000), mice with single anesthesia (Mann-Whitney-U-Test: U = 114.000, p = 0.139), and mice with repeated anesthesia (Mann-Whitney-U-Test: U = 146.000, p = 0.649) (Table 8).

## Discussion

Within the context of refinement, the aim of this study was to investigate whether repeated anesthesia with the combination of ketamine and xylazine (KX) has a higher impact on well-being of adult female and male C57BL/6JRj mice than single anesthesia with KX. Body weight, corticosterone (metabolite) concentrations, and behavioral parameters such as nest building, MGS, food intake, anxiety-related behavior, and motor activity were compared between single and repeated anesthesia. Furthermore, parameters of anesthesia monitoring, e.g. phases of anesthesia and vital parameters, were recorded.

When investigating the impact of repeated anesthesia with KX on well-being of mice, it has to be considered that these effects can be caused by the procedure itself, i.e. restraining and injection stress when administering the drug, bad experience due to the loss of control during anesthesia and the recovery period, but can also be the result of direct pharmacological drug effects. In our study, the whole process of anesthesia including pharmacological effects, as well as restraining and injection stress was of interest.

The main findings of our study were that repeated anesthesia with KX caused short-term effects on MGS scores in the immediate post-anesthetic period and on well-being of female mice, indicated by longer lasting effects on trait anxiety-related behavior. The rapid jerky dorsal-ventral head movements may suggest more intense psychogenic effects after repeated *versus* single administration of KX. However, MGS scores and well-being were also slightly impaired in the immediate post-anesthetic period after single KX anesthesia: compared to control animals, MGS scores increased and, in females, higher trait anxiety levels were observed. Short-term changes in food intake, depending on sex, and FCM excretion pointed towards mice being likely to suffer from distress after single anesthesia, whereas they seemed to habituate to the stress induced by the repeated procedure of KX anesthesia. Additionally, we observed a shortened duration of KX anesthesia after repeated administration which points towards a narcotic tolerance development to KX.

### Free exploratory paradigm

Repeated anesthesia with KX increased the latency to explore in the free exploratory paradigm in females, but not in males, indicating higher trait anxiety levels in female mice. This is in line with investigations of repeated ketamine administration revealing opposite behavioral effects in female and male mice, indicated by anxiogenic effect exertion in the open field, a test for state anxiety, in females [16]. In contrast, it had been shown that female rats did not display higher state anxiety levels in the elevated plus maze test following repeated administration of ketamine [42]. For α_2_-adrenoceptor-agonists, anxiolytic properties were described [43]. When determining causes of trait anxiety increase, the injection stress as a confounding factor for anxiety-related behavior must also be considered. Intraperitoneal injection of saline can cause an anxiogenic effect in the elevated plus maze test [44].

It is known that the gonadal hormones play a critical role in the effect mechanism of ketamine [45], which may explain the sex-dependent behavioral effects observed in mice. In our study, single anesthesia increased trait anxiety-related behavior at day eight and repeated anesthesia at day one and day eight after the last administration in females. This is in line with deficits induced by a chronic unpredictable stress model still being reversible in male rats seven days after a single administration of ketamine [46]. As we only conducted the free exploratory paradigm after the last anesthesia, we could not determine whether trait anxiety-related behavior was also increased after the second, third, fourth or fifth anesthesia. In order to investigate whether female mice receiving repeated anesthesia experience higher trait anxiety short-term or long-term, further investigations including tests for anxiety-related behavior after each anesthesia are warranted.

### Mouse Grimace Scale and nest building

Short-term negative effects of isoflurane anesthesia on the Mouse and Rat Grimace Scale were previously reported [4, 47, 48]. Our study is the first to show a comparable effect following anesthesia with KX in mice. At 150 min after single KX anesthesia as well as after the last repeated KX anesthesia, the MGS was higher in comparison to control levels. Photographs taken two days later showed no alteration in facial expression anymore. A pharmacological effect on the MGS cannot be excluded, as the serum half-life of ketamine is approximately 13 min in mice [49] and that of xylazine is 1.3 h in young rats, when administered intraperitoneally [50]. Latter effect is not known in mice, but elimination half-life presumably is shorter in mice than in rats [50–52]. The cataleptic effect of ketamine [10, 12], which increases the muscle tonus, may explain higher MGS scores for whisker change. Another explanation for the increase in MGS scores could be nausea. Mice do not vomit, but can suffer from post-operative nausea [53]. However, we could not determine the exact time point of their facial expression returning to control levels, as our protocol did not allow for an earlier time point to take photographs of the mice.

The effects of KX anesthesia itself on the MGS must be taken into account when applying the MGS to assess pain at early time points, as described previously for isoflurane in mice and rats [4, 47, 48].

To the authors’ knowledge, nest building has not been investigated following KX anesthesia to date. It is only present when important needs of the mice are met and thus can serve as an indicator of well-being [54]. However, nest building can also be impaired by hippocampal lesions [55]. In the present study, we demonstrated that neither single nor repeated KX anesthesia impaired nest scores according to Deacon [56] one day after anesthesia (i.e. 2 h after lights turned on). It should be noted that both substances, ketamine and xylazine, evoke hypothermia in rodents [57, 58], which in turn triggers building of high and complex nests to decrease the amount of radiated heat and, thus, alleviate thermal discomfort [59]. In our previous study, we found that neither single nor repeated anesthesia with isoflurane impaired nest scores [4] obtained by using the same nesting protocol [56].

### Corticosterone and its metabolites

The influence of the circadian rhythm on FCM excretion was excluded [60] by collecting samples over the 24 h post-anesthetic period [4]. In general, HPA axis activity and corticosterone release are higher in female rodents than in males (S1 Table), which was shown for baseline [61] as well as in response to stress insults [62]. For statistical analysis, we calculated the percentage change relative to baseline values. Interestingly, at day one after the last anesthesia, the percentage change of FCM levels was higher in male than in female groups with statistical significance after single anesthesia. In addition, male mice receiving single anesthesia showed higher percentage values in comparison to controls. The increase in FCM values following single anesthesia reflected higher acute stress levels.

The same method for FCM analysis was previously used to identify a correlation between an increase in FCM levels and various stressors in mice [4], e.g. train-induced vibrations [63], oral gavage [64], and blood sampling [65].

Similar observations to our study were evident in pigtailed macaques with elevated urinary free cortisol levels after ketamine sedation [29]. The effect of KX anesthesia on plasma corticosterone concentrations seems to vary depending on sex and species. Saha et al. found a decrease in plasma levels of corticosterone in male rats following KX anesthesia [17], whereas Illera et al. determined no changes in female rabbits [66]. Accordingly, FCM concentrations in mice receiving single anesthesia were not primarily due to the pharmacological effects of ketamine or xylazine, but rather a result of restraining and injection stress. When a mouse is restrained and injected for the first time, a clear stress response can be measured, i.e. by heart rate and plasma corticosterone increase [23]. Mice require at least two weeks to habituate to stress caused by repeated injections, as shown for C57BL/6J [67]. Our results of FCM analysis suggested that mice habituated to the procedure of anesthesia including restraining, injection, and the recovery period. Stress may be minimized by administering isoflurane anesthesia prior to the unpleasant injection of KX as described for rats [9]. In our previous study, we demonstrated that repeated administration of isoflurane for 45 min did not significantly elevate the percentage change of FCM levels relative to baseline levels when compared to single anesthesia or controls [4].

In contrast to FCM levels, hair corticosterone concentrations, which serve as a retrospective biomarker to reflect long-term HPA axis activity in humans [68] and rodents [69], did not differ between study groups. In our previous study, we also did not find any effects on hair corticosterone levels following single or repeated anesthesia with isoflurane [4]. However, studies on social defeat [70] and social instability [71] in mice have shown that hair corticosterone concentrations increase when the animals are confronted with stress.

To sum up the results of FCM and hair corticosterone concentrations, single anesthesia seemed to cause a short-term HPA axis response in mice, but did not seem to cause chronic stress.

### Food intake and body weight

Male mice anesthetized only once ingested less food in comparison to male mice receiving repeated anesthesia, which indicates distress following single anesthesia. This was also observed in pigtailed macaques with appetite suppression after ketamine sedation [29]. Ketamine disturbs the circadian rhythm and, therefore, may result in a reduced food intake [14, 72]. Moreover, an increase in stress levels, as indicated by elevated FCM levels in male mice after single KX anesthesia, can explain appetite suppression [29]. Lenglos et al. reported that acute stress decreased 24-h food intake in rats of both sexes [73]. Moreover, the injection of KX increases blood glucose levels in C57BL/6 mice of both sexes [17, 74–76], which subsequently can also reduce appetite and food intake.

Despite the changes in food intake, body weight did not statistically differ between controls, single, and repeated anesthesia. For both anesthesia regimens, body weight decreased two days after the last anesthesia, but increased over the following days. As control mice of both sexes also lost weight at this time point, body weight appeared to be influenced by single housing required during the 24-h observation period, although all efforts were made to reduce distress (used bedding material; visual, acoustic and olfactory contact; duration of single housing kept to a minimum).

A similar body weight loss was present in control and male rats administered with a single anesthesia of ketamine, xylazine, and acepromazine [27]. Dholakia et al. reported that male CD-1 mice at four to six weeks of age also lost an average of 1.6% in body weight by day two post-anesthesia with ketamine-xylazine-lidocaine [26]. Interestingly, in our study, female and male mice anesthetized only once lost nearly twice as much body weight than those anesthetized repeatedly. The lower loss in body weight after repeated anesthesia may be due to habituation to the anesthesia procedure, as suggested by the lower FCM levels. Also, the higher body weight loss after single anesthesia may be due to forced diuresis. Xylazine increases urine production and excretion [77]. This effect may be stronger after single anesthesia since repeated administration of xylazine can result in a its tolerance, as also shown for its analgesic effect [78].

### Recovery period

KX anesthesia produced stereotypic behavior (i.e. rapid jerky dorsal-ventral head movements while being stationary [39]) during the recovery period. The NMDA receptor antagonist ketamine is known to cause stereotypies, ataxia, and hyperlocomotion in rats [79] and mice [80]. Behavioral patterns such as head weaving, ataxia, infrequent circling, and reciprocal forepaw treading were described in rats following administration of an uncompetitive NMDA receptor antagonist [81]. Interestingly, ketamine also enhances the head twitch response mediated by the 5-HT_2_ (serotonin, 5-Hydroxytryptamine) receptor in mice indicating a glutamatergic modulation of serotonergic function at the postsynaptic 5- HT_2_ receptor [82, 83]. More recently, the affinity of the NMDA receptor antagonists phencyclidine and its congener ketamine for both 5-HT_2_ and dopamine D_2_ receptors was proven [84]. Thus, it is possible that the rapid jerky dorsal-ventral head movements we observed in our study are 5-HT_2_-mediated head twitch responses which serves as behavioral assay for hallucinogen-like effects [85]. This behavior pattern was exacerbated when KX was repeatedly administered, suggesting an increase in psychogenic effects, which has been observed in frequent ketamine users displaying greater dissociative and delusional symptoms [86].

### Anesthesia

With repeated administration of KX the overall duration of anesthesia decreased in mice of both sexes with a shorter duration in females. This is in line with findings in rats [28, 31] and rhesus macaques [87] repeatedly anesthetized with KX. A daily intraperitoneal treatment with low dose-ketamine over 10 days in rats resulted in a faster metabolism of ketamine so that circulating and brain levels of ketamine and its N-demethylated metabolite decreased more rapidly [31]. Consequently, Livington and Waterman concluded that narcotic tolerance to ketamine in rats can be explained by an increase in hepatic metabolism [31]. We suggest that the decrease in overall anesthesia duration and surgical tolerance in mice with repeated anesthesia may also be associated with similar mechanisms. In addition, as stated previously, a tolerance can also be developed against xylazine [78].

In our study, surgical tolerance was reached in all mice anesthetized only once but KX failed to produce surgical tolerance in a total of five mice when the sixth anesthesia was performed. Thus, we could verify previous findings of Arras *et al*. [8]. Since higher doses and repeat-bolus dosing can prolong the recovery period and cause severe respiratory depression [21], which increases the risk of mortality [8, 88], all mice received only one of KX dose in our study–even if they did not reach surgical tolerance.

KX anesthesia depressed the heart and respiratory rate in the present study, as expected from literature [89], however, without difference between single and repeated anesthesia. This is consistent with the effect of repeated KX anesthesia on heart rate in rats, which underwent the same anesthesia protocol as mice of our study [28]. Although KX anesthesia is known to cause hypoxemia, oxygen administration is still not common laboratory practice [90, 91]. As our study was designed to model a common KX anesthesia protocol, oxygen was not supplied [91]. Consequently, all mice in our study suffered hypoxemia. In humans, hypoxemia can elicit the subjective feelings of malaise, headache, dizziness, fatigue, and insomnia (e.g. when suffering from acute mountain sickness) [92]. If we expect similar responses in mice, hypoxemia will impair well-being, which emphasizes the need of oxygen administration during KX anesthesia. Dittmar et al. pointed out the importance of pre-oxygenation prior to administration of KX [9]. Further, oxygen insufflation should be continued until animals reach a stable anesthesia state [9]. In contrast to anesthesia with KX, we measured high values of oxygen saturation (> 98%) during inhalation anesthesia with isoflurane (in 100% oxygen) in our previous study [4].

### Limitation

Limitations of this study are the use of this exact anesthesia protocol and dosage in solely one particular mouse strain. Firstly, strain-dependent stress responses and tolerances towards KX dosages are known [8, 67]. Besides the KX dose, anesthesia protocols vary in duration of anesthetic episodes, number of consecutive anesthetic episodes, and intervals between the anesthetic episodes. In the present study, we followed an anesthesia protocol described by Albrecht et al. [28] and used a dosage of 80 mg/kg ketamine and 16 mg/kg xylazine [35]. The effects of repeated KX anesthesia on well-being of mice revealed here only refer to this anesthesia protocol and cannot be transferred to other protocols. The present study can serve as a basis for further investigations of different anesthesia protocols (e.g. anesthesia episodes at an interval of 1–2–4–8 weeks). The authors would like to encourage the implementation of the methods described here in studies with repeated anesthetization to investigate the effects of the respective anesthesia protocol on the well-being of mice. The methods can easily be integrated in a planned study.

Since control animals did not receive a vehicle injection, we cannot determine whether effects observed in our study were due to pharmacological KX effects or whether they were caused by restraining and injection stress. Control animals were not injected with a vehicle solution because our study should investigate the impact of the entire KX injection anesthesia procedure, also including restraint and injection besides pharmacological effects. If control mice had received a vehicle injection, we could have only examined the pharmacological effect of KX.

The injection site was not histologically examined. Depending on the route of administration, KX can cause tissue damage at the injection site. Whilst intramuscular injection of KX causes tissue damage [28, 93, 94], subcutaneous injection leads to mild edema with mixed infiltration of neutrophils and lymphocytes [95] at the injection site in rodents. The same also applies to the intraperitoneal route, which can be accompanied by local tissue damage and an increase in serum creatine kinase concentrations in rats [25]. Wellington et al. also demonstrated that rats expressed momentary pain and discomfort after intraperitoneal injection of KX [25]. Tissue damage may be minimized by using small and short needles as well as by injection site alternation [36].

Another limitation is the single housing in new cages during the 24 h observation period, which presumably caused distress in mice indicated by a loss in body weight. However, as controls underwent the same tests, the impact of single housing on the results was considered.

For severity classification of a procedure, the duration of negative effects on well-being is essential. Depending on the duration (short-term or long-term), mild pain, suffering or distress may be classified as mild or moderate: “Procedures on animals as a result of which the animals are likely to experience short-term mild pain, suffering or distress, as well as procedures with no significant impairment of the well-being or general condition of the animals shall be classified as ‘mild’. Procedures on animals as a result of which the animals are likely to experience short-term moderate pain, suffering or distress, or long-lasting mild pain, suffering or distress as well as procedures that are likely to cause moderate impairment of the well-being or general condition of the animals shall be classified as ‘moderate’ [1].” Therefore, more observations are warranted to clearly determine whether repeated KX anesthesia only causes short-term mild effects (severity category “mild”) or whether it causes long-term mild effects (severity category “moderate”).

### Conclusion

Both single and repeated anesthesia with KX for six times every three to four days increased MGS scores in adult C57BL/6JRj mice of both sexes during the immediate post-anesthetic period, whilst repeated anesthesia additionally exacerbated psychogenic effects. Effects on anxiety were sex-dependent with longer lasting effects on trait anxiety levels in female mice after both single and repeated anesthesia.

All in all, negative effects on well-being did not accumulate in repeatedly anesthetized mice since stress and anxiety levels were comparable to mice anesthetized only once. Hence, effects of both single and repeated anesthesia can be considered mild. Nevertheless, the duration of increased stress and anxiety levels, which needs to be considered in severity classification, may be longer in mice repeatedly anesthetized and should be examined in further investigations.

## Supporting information

S1 Methods Behavioral parameters and stress hormone (metabolite) analyses. (PDF)

S1 Table Fecal corticosterone metabolites (FCM) and hair corticosterone. Data are given as median (M) and interquartile range (IQR). FCM, fecal corticosterone metabolites. ^a^1 male mouse of the repeated anesthesia group was excluded from the statistics concerning hair corticosterone because we did not collect enough sample material.
(GIF)

S2 Table Raw data–fecal corticosterone metabolites. (XLSX)

S3 Table Raw data–hair corticosterone. (XLSX)

S4 Table Raw data–vital parameters during anesthesia. (XLSX)

S5 Table Raw data–phases of anesthesia. (XLSX)

S6 Table Raw data–recovery period. (XLSX)

S7 Table Raw data–Mouse Grimace Scale. (XLSX)

S8 Table Raw data–nest building. (XLSX)

S9 Table Raw data–home cage activity. (XLSX)

S10 Table Raw data–rotarod test. (XLSX)

S11 Table Raw data–free exploratory paradigm. (XLSX)

S12 Table Raw data–food intake. (XLSX)

S13 Table Raw data–body weight. (XLSX)
