# Supplementary material for: Impact of repeated anesthesia with ketamine and xylazine on the well-being of C57BL/6JRj mice
Source: PLoS One. 2018 Sep 19;13(9):e0203559. doi: 10.1371/journal.pone.0203559 (PMC6145541; doi:10.1371/journal.pone.0203559)
Supplement: S1 Methods — (PDF) [file pone.0203559.s001.pdf]

Behavioral parameters

Mouse Grimace Scale (MGS):

MGS scores [1] were obtained by photographs taken in a custom-made box (22 × 29 × 39 cm, consisting of three white walls and one clear wall, with 0.5 cm bedding material including soiled bedding) two days prior to first anesthesia (baseline), 150 min and two days after the last anesthesia [2]. A high definition camera (Canon EOS 350D, Canon Inc., Tokyo, Japan) was used to continuously take about 30–40 photographs for each time point within 1 - 2 min [2].

Blurry photographs or photographs that show mouse faces from perspectives other than frontal or lateral view were discarded [1, 2]. One photograph was randomly selected for each time point [2]. The photograph was cropped to only display the head of the mouse so that the body position was not visible [1]. According to Langford et al. [1], five facial action units (i.e. orbital tightening, nose bulge, cheek bulge, ear position, and whisker change) were scored on a scale from 0 to 2 (0 = not present, 1 = moderately present, 2 = obviously present).

Four blinded persons analyzed the photographs independently. For each scorer, the mean of the five facial action units was calculated. Then, the mean MGS difference score was calculated between the baseline MGS score and the MGS score at 150 min as well as two days after last anesthesia, respectively [1]. The mean MGS difference scores obtained by the four persons were averaged for each mouse for further statistical analysis.

Nest building

Nest building behavior is only present when important needs of the mice are met andcan thus serve as indicators of well-being [3]. The nests were scored using a modified protocol developed by Deacon [4]. A nestlet (Ancare, Bellmore, NY, USA; UK agent: Lillico, Betchworth, UK) with an exact weight of 2.0 g was placed in the middle of the cage (Makrolon type III, 420 × 260 × 150 mm) containing 0.5 cm thick bedding material. In order to reduce distress caused by a new environment, used bedding material without feces from the home cage was scattered on top of the new bedding. No further environmental enrichment items were provided.

Two hours after the light was turned on, the nests were assessed on a 5-point scale (1 = more than 90 % of the nestlet intact; 2 = 50–90 % intact; 3 = 50–90 % shredded nestlet; 4 = more than 90 % shredded but flat nest, i.e. less than 50 % of its circumference being higher than the body height of a curled-up mouse; 5 = more than 90 % shredded and high nest, i.e. more than 50 % of its circumference being higher than the body height of a curled-up mouse) [4]. Untorn nestlet pieces of approximately 0.1 g were weighed.

Home cage activity

Home cage activity was evaluated by InfraMot (TSE systems, Bad Homburg, Germany) during the dark period for 12 h (6:00 p.m. to 6:00 a.m.). An infrared sensor was mounted on the gridded cage top and the number of impulses per minute was recorded. For analysis, impulse intervals of 10 min were used and the area under the time curve (AUC) [impulses/10 min] was calculated.

Rotarod test

An accelerating rotarod (TSE systems, Bad Homburg, Germany) was used to evaluate motor coordination and balance. The mice were gently placed on the rotating drum at a speed of four rounds per minute. The speed of the rotarod accelerated to 40 rounds per minute. The latency

[s] to loose balance and fall off the rotating drum was measured. Some mice held on and rode around the rotarod a few times before they lost their balance. For those mice, the time until they finally fell off the rotarod was recorded. Mice performed four trials at two days after the last anesthesia, with a maximum of 300 s and 30-min intertrial rest intervals [5, 6]. Trials one to three served as training and trial four as the test trial.

Free exploratory paradigm

The free exploratory paradigm investigates trait anxiety-related behavior [7]. The gridded cage top was placed in the cage at an angle of 45° on the longer side of the home cage. The latency to first exploration [s] (with all four paws on the lid) within 10 min was observed.

Food intake and body weight

Food intake of the standard food diet [g] was manually measured over a period of 24 h. The cage side beneath the food unit was carefully scanned for tiny pieces of food pellets. For analysis, the amount of food intake per g body weight was calculated.

The body weight [g] was regularly controlled over the experimental period. For analysis, the body weight of day 0, day 2, day 9, and day 14 after last anesthesia was used.

Analysis of corticosterone and its metabolites

Fecal corticosterone metabolites (FCM) with a 5α-3β,11β-diol structure in feces and hair corticosterone were measured since these sampling techniques are non-invasive. In our study, FCM indicate acute stress during the 24 h post-anesthetic period and hair corticosterone reflects chronic stress. Fecal samples were collected one day prior to first anesthesia (baseline), and one as well as eight days after the last anesthesia; hair samples were taken two days prior to first anesthesia (baseline) and at the end of the study, when hair had regrown. Since there are individual differences in baseline values, the percentage change [%] relative to baseline was calculated for each mouse.

Fecal corticosterone metabolites (FCM)

The mice were single-housed for a period of 24 h. In order to prevent distress due to a new cage, used bedding material without feces from their home cage was scattered on top of the new bedding. At the end of the 24 h period, all dry fecal pellets were collected using forceps. Wet pellets contaminated with urine were eliminated.

FCM were extracted from the 24-h-bulk samples according to Palme et al. [8]. Briefly, fecal samples were dried at a temperature of 60–70 °C and homogenized by a mortar. An aliquot of 0.05 g was shaken with 1 ml of 80 % methanol for 30 min on a multi-vortex. After centrifugation (2500 x g, 15 min), 0.5 ml of supernatant was pipetted it on an Eppendorf cup. Before and after extraction, the samples were stored at –80 °C. The samples were analyzed for corticosterone metabolites using a 5α-pregnane-3β,11β,21-triol-20-one enzyme immunoassay as described and validated for mice by Touma et al. [9, 10]. Intra- and interassay coefficients of pool sample variation were below 10 % and 15 %, respectively.

Hair corticosterone

Approximately 7.5 mg of hair () from the left hind leg (lateral side of the thigh, approximately 1.5 cm^2) was shaved with an electric shaver for small animals (Aesculap Isis GT 420, Suhl, Germany). This location was chosen since the sensor of the pulse oximeter was attached to the left hind leg during anesthesia. Both samples (baseline, end of the study) were taken from the same location in order to obtain information on long-term corticosterone secretion reflecting chronic stress. The shaver was cleaned before and after use with paper tissues or Q-tips soaked in water. Hair corticosterone [pg/mg] was analyzed by liquid chromatography-mass spectrometry in the laboratory of Prof. Kirschbaum, Department of Psychology, Technische Universität Dresden, Germany, as described previously [11].

Literature

1. Langford DJ, Bailey AL, Chanda ML, Clarke SE, Drummond TE, Echols S, et al. Coding of facial expressions of pain in the laboratory mouse. Nat Methods. 2010;7(6):447-9.
2. Hohlbaum K, Bert B, Dietze S, Palme R, Fink H, Thöne-Reineke C. Systematic assessment of well-being in mice for procedures using general anesthesia. J Vis Exp. 2018a;(133):e57046.
3. Jirkof P. Burrowing and nest building behavior as indicators of well-being in mice. J Neurosci Methods. 2014;234:139-46.
4. Deacon RM. Assessing nest building in mice. Nat Protoc. 2006;1(3):1117-9.
5. Paylor R, Spencer CM, Yuva-Paylor LA, Pieke-Dahl S. The use of behavioral test batteries, II: effect of test interval. Physiol Behav. 2006;87(1):95-102.
6. Yonezaki K, Uchimoto K, Miyazaki T, Asakura A, Kobayashi A, Takase K, et al. Postanesthetic effects of isoflurane on behavioral phenotypes of adult male C57BL/6J mice. PloS One. 2015;10(3).
7. Bert B, Schmidt N, Voigt JP, Fink H, Rex A. Evaluation of cage leaving behaviour in rats as a free choice paradigm. J Pharmacol Toxicol Methods. 2013;68(2):240-9.
8. Palme R, Touma C, Arias N, Dominchin MF, Lepschy M. Steroid extraction: Get the best out of faecal samples. Wien Tierarz Monats. 2013;100(9-10):238-46.
9. Touma C, Sachser N, Mostl E, Palme R. Effects of sex and time of day on metabolism and excretion of corticosterone in urine and feces of mice. Gen Comp Endocrinol. 2003;130(3):267-78.
10. Touma C, Palme R, Sachser N. Analyzing corticosterone metabolites in fecal samples of mice: a noninvasive technique to monitor stress hormones. Horm Behav. 2004;45(1):1022.
11. Gao W, Stalder T, Foley P, Rauh M, Deng H, Kirschbaum C. Quantitative analysis of steroid hormones in human hair using a column-switching LC-APCI-MS/MS assay. J Chromatogr B Analyt Technol Biomed Life Sci. 2013;928:1-8.
