# Supplementary figures and images for: Impact of repeated anesthesia with ketamine and xylazine on the well-being of C57BL/6JRj mice
Source: PLoS One. 2018 Sep 19;13(9):e0203559. doi: 10.1371/journal.pone.0203559 (PMC6145541; doi:10.1371/journal.pone.0203559)

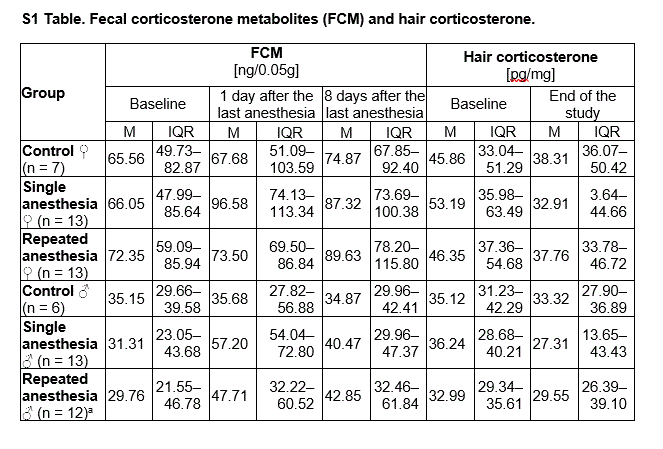

Supplement: S1 Table — Data are given as median (M) and interquartile range (IQR). FCM, fecal corticosterone metabolites. a1 male mouse of the repeated anesthesia group was excluded from the statistics concerning hair corticosterone because we did not collect enough sample material. (GIF) [file pone.0203559.s002.GIF]
